# Supplementary material for: Racial differences in testing for infectious diseases: An analysis of jail intake data
Source: PLoS One. 2023 Dec 20;18(12):e0288254. doi: 10.1371/journal.pone.0288254 (PMC10732427; doi:10.1371/journal.pone.0288254)
Supplement: Attachment — Submitted filename: PLOS ONE review.docx [file pone.0288254.s003.docx]

PLOS ONE review comments

General:

-While it’s interesting to look at restrictive housing and psychiatric illness, you haven’t addressed that content in the Discussion. I suggest you consider cutting that info or else explicitly interpreting that information and discussing the implications.

Abstract:

-I find this sentence unclear: “We hope our collaborative efforts with the jail professionals can encourage other cross-disciplinary investigations.”

Introduction:

-paragraph 2 (and throughout)- I’m interested to see the use of the term “minoritized” rather than “racialized”- given the explicit focus on race, why not use “racialized”?

-paragraph 2- I think the content in this paragraph from “Disparities in access to HIV and HCV testing…” onward seems unrelated to the content in the first half of the paragraph, including the initial sentence. Consider splitting up the paragraph or revising the initial sentence to improve coherence/flow.

Methods:

-line 73- is the language used to ask people if they want testing actually “HCV” or “hepatitis C”? I assume not everyone would know the acronym HCV and so wonder how accessible this question would be.

-line 74- is the “contracted community health group” the MassDPH? I find this unclear. May need to clarify consent+specimen collection vs. testing…

-line 79- would be nice to know who does case management and contact tracing, partly to show the value of the jail-based testing

-lines 79-83- I understand that there are multiple opportunities to request testing, but can you clarify whether all testing is conducted by MassDPH? i.e. are the data presented comprehensive for all those tested?

-lines 90-91- Should describe whether data on race/ethnicity are self-reported vs. assigned, and who collects these data (e.g. a correctional officer?).

-93-96-why only look at intake data on psychiatric history, recognizing that some people will be diagnosed with or initially report psychiatric illness subsequent to admission? Good to clarify and/or report as a limitation.

-90-90 vs. 107-108- why are the cats different in these two sections? Is this the difference between the primary data collection and the dataset?

-lines 116-118- “People who did not request testing at intake, but completed testing, were classified as having completed testing because they likely requested testing at another time during their incarceration.” Should this say “were classified as having *requested* testing”?

Results

-lines 125-127- Excluding those with any missing data may bias the findings. Why not include all persons and report missing data whenever possible? Especially since this seems to be a fairly large number of people with any missing data (>400 in each cohort) and missingness is likely non-random.

-line 156-I would provide absolute numbers for those with and without psychiatric illness, in addition to AOR data. Also should use the same language throughout, i.e. is it “mental health history,” “psychiatric illness history,” or “treated psychiatric illness history”? Same issues in Tables…

-line 161-162- Aren’t you looking at receipt of testing for all persons and not just those who opted in to testing? i.e. are you looking at receipt of testing conditional on opting in? This isn’t what you describe in the Methods…I think both are important but need to clarify which you are assessing and line up content in the Methods+Results accordingly.

-lines 164-165- Could this be an issue of tests vs. people tested? i.e. 449/479. Also, I don’t think you talked about accessing data on tests performed from DPH in Methods- can you reconcile/clarify this?

-line 170- You mentioned the conflation of race/ethnicity in the categories, and yet you are using the word *race* in the text. Why not consistently use race/ethnicity throughout?

-line 174, line 180- I think the language re: the second outcome is unclear here- sounds like you are looking at receiving the test result vs. being tested, which is different from the definition of second outcome in line 104. Related to this, consider whether helpful to revise the language throughout to say “Obtained testing” or “Completed testing” rather than “Received testing.” Same issue in Table 3 (“received results”).

Discussion

-line 183-185- compared with…

-line 186- I wonder if it would be more appropriate to define the first outcome as “opting in to testing” vs. “requesting testing.” Requesting suggests greater initiative on the part of the person in custody than what is actually happening. Also may be good to clarify whether those who requested testing at a time other than intake are included in cohort 1- this isn’t clear to me. (Line 163 says “The remaining completed tests (111) were done at a time outside of intake.” but I’m not sure if this is about the timing of testing vs. opting in/requesting).

-lines 188-189- “Our results show that there is still much work to be done towards the goal of getting people who are incarcerated and detained access to infectious diseases testing” Can you clarify what you mean here? I think the data don’t clearly show that access is the issue, vs. acceptability…

-lines 193-204-Can you be explicit about what you think the goal is for testing? Is it universal testing in custody? testing where there is an unmet need for testing? Also recommend you note that it is a limitation that you don’t have access to data on community-based testing (to look at recent testing) or data on testing indication (e.g. IDU, sexual behaviours, etc.).

-lines 205-212-You have data on dates of incarceration- couldn’t you do some simple stratified analyses of whether/how length of time in custody is associated with testing as a supplementary analysis? Seems odd to me to speculate about this when you have the data to easily look at it.

-line 218- acceptability or acceptance?

-line 248-249- Wouldn’t some of these errors lead to testing not being conducted, e.g. an error in specimens not being sent to MassDPH?

-lines 258-259- I don’t see why you can’t look at who had HIV and HCV since you said you had access to those data in the Methods. Then you could remove those people from the analysis and focus on those eligible for screening.

-lines 267-268- The assessment of “ever tested” isn’t clear in the Methods and I think this is problematic. I think you should consider whether to run analyses for one admission (random, most recent, etc.) rather than by person. Alternatively, you should clarify what you did and why in the Methods.

-273-277-Given the substantial limitations of use of available administrative data to understand why people aren’t being tested, I think it makes sense to talk about additional work, e.g. engaging with health care workers (those offering opt in testing, those conducting testing) and people in the jail to understand the issues better, which could be through qualitative research or engagement with these groups to support interpretation. What exactly the “improved protocols” would be is very unclear based on the study findings.

Tables:

-Table 1- rephrase “ever asked about Infectious Disease Testing” to “Offered opt in testing for HIV/HCV/syphilis”?

-Table 1- final rows- add total tested

-Table 2-define “restrictive housing” below the table

-Tables 2 and 3-restrictive housing and psychiatric illness are not sociodemographic characteristics- revise title
